# The role of glucocorticoid action in the pathophysiology of the Metabolic Syndrome

**DOI:** 10.1186/1743-7075-2-3

**Published:** 2005-02-02

**Authors:** Minghan Wang

**Affiliations:** 1 Department of Metabolic Disorders, Amgen Inc., Thousand Oaks, CA, USA

## Abstract

Glucocorticoids are stress hormones that modulate a large number of physiological actions involved in metabolic, inflammatory, cardiovascular and behavioral processes. The molecular mechanisms and the physiological effects of glucocorticoids have been extensively studied. However, the involvement of glucocorticoid action in the etiology of the Metabolic Syndrome has not been well appreciated. Recently, accumulating clinical evidence and animal genetics studies have attracted growing interest in the role of glucocorticoid action in obesity and insulin resistance. This review will discuss the metabolic effects in the context of glucocorticoid metabolism and establish the association of glucocorticoid action with the features of the Metabolic Syndrome, especially obesity and insulin resistance. Special discussions will be focused on corticosteroid-binding globulin and 11β-hydroxysteroid dehydrogenase type 1, two proteins that mediate glucocorticoid action and have been implicated in the Metabolic Syndrome. Due to the complexities of the glucocorticoid biology and the Metabolic Syndrome and limited space, this review is only intended to provide a general link between the two areas with broad rather than in-depth discussions of clinical, pharmacological and genetic findings.

## Introduction

Insulin resistance and hyperinsulinemia are often associated with a group of risk factors such as obesity, dyslipidemia, hypertension and impaired glucose tolerance. This cluster of metabolic abnormalities, first defined as Syndrome X by Reaven in 1988 [1] and supported by additional evidence [2,3], is now more often referred to as the Metabolic Syndrome and has been increasingly recognized as important risk factors for coronary artery disease (CAD). The point of view became institutionalized and although the National Cholesterol Education Program's Adult treatment Panel III (ATP III) and the World Health Organization (WHO) have slightly different definitions [4-6], the Metabolic Syndrome is consistently characterized by a collection of metabolic abnormalities such as insulin resistance, obesity, dyslipidemia, hyperglycemia, and hypertension [7]. Not all of the disorders in the Metabolic Syndrome may be observed in the same individual. Most people with the syndrome have insulin resistance that could lead to glucose intolerance and diabetic hyperglycemia. Although the mechanisms underlying the pathogenesis of the Metabolic Syndrome are not exactly clear, obesity, insulin resistance and other independent factors such as vascular and immunologic origins appear to be involved [7]. The prevalence of the Metabolic Syndrome is more than 20% among the US adults adjusted for age [8], which is far greater than observed in an earlier study with European participants at least partly due to differences in the criteria used to define the condition [9]. Increased cardiovascular and mortality risks are associated with the Metabolic Syndrome [10]. The condition is usually managed with pharmaceutical agents for correcting dyslipidemia, anti-hypertensives, and insulin sensitizing agents or a combination of the above. Most existing agents only treat individual metabolic abnormalities. To date, no single agent can ameliorate all the features of the Metabolic Syndrome. There is an increasing need for novel agents to treat multiple abnormalities of the syndrome.

Glucocorticoid (GC) excess has been linked to clinical observations associated with the Metabolic Syndrome. In Cushing's syndrome [11], increased secretion of GCs largely due to pituitary adenoma leads to central obesity, hypertension, hyperlipidemia and glucose intolerance, a group of metabolic abnormalities reminiscent of the Metabolic Syndrome. Correction of hypercortisolism by transsphenoidal surgery at least normalizes blood pressure [12,13]. In addition, clinical administration of GCs to treat acute and chronic inflammatory diseases has been associated with metabolic adverse effects such as hypertension, obesity, hyperlipidemia and insulin resistance as seen in the Metabolic Syndrome [14-16]. These clinical findings suggest that GC action could play a role in the pathophysiology of the Metabolic Syndrome.

## GC metabolism and action

Cortisol, the principal active GC in humans, is secreted by the adrenal gland and is converted to cortisone, the inert GC, primarily in kidney [17-19]. Two isozymes of 11β-hydroxysteroid dehydrogenase (11β-HSD) are responsible for the tissue-specific interconversion of cortisone and cortisol at the endoplasmic reticulum: type 1 and 2 (11β-HSD1 and 11β-HSD2) [20]. The two isozymes are products of two different genes and have distinct tissue distributions, with 11β-HSD1 expressed primarily in liver, adipose, kidney and brain and 11β-HSD2 mainly in kidney and salivary glands [20]. 11β-HSD1 converts inactive cortisone to cortisol in human or inactive 11-dehydrocorticorsterone (11-DHC) to corticosterone in rodents and 11β-HSD2 catalyzes the opposite reaction. Bidirectional activities (both reductase and dehydrogenase) have been observed with 11β-HSD1 *in vitro *but it is mainly a reductase *in vivo *[21]. Since GC action is largely mediated by the ligand-induced activation of the GC receptor (GR), the local concentration of cortisol (or corticosterone) dictates GR activation. In tissues such as liver and adipose where 11β-HSD1 is expressed, there are two sources for cortisol (or corticosterone) accumulation: the fraction produced by 11β-HSD1 within the tissue and that from the plasma by diffusion. Obviously, 11β-HSD2 activity is responsible for reducing the cortisol level in kidney [17-19]. In addition, cortisol metabolism in liver is part of the balance maintaining the tissue-specific cortisol concentration.

The circulating cortisol level undergoes circadian variations peaking in the early morning at approximately 800 nM and reaching a nadir of about 200 nM at midnight [22]. The plasma cortisone level is much lower and shows no significant circadian rhythm [22]. The salivary cortisol level exhibits a similar trend of diurnal rhythm [23]. Rodents housed under 12-h light, 12-h dark illumination conditions exhibit an opposite pattern of circadian variation with lowest circulating corticosterone levels in the early morning and the peak concentration at the light/dark transition phase before declining to nadir [24]. The plasma GC level is regulated by the activity of the hypothalamic-pituitary-adrenal (HPA) axis, a neuroendocrine feedback circuit that can be activated by physiological stimuli such as stress [25]. Plasma cortisone is largely in the free unbound form but approximately 6% cortisol is bound to albumin and 90% is bound to corticosteroid-binding globulin (CBG), a protein synthesized in liver and secreted in blood [26,27]. Since only free cortisol is active, CBG binding may restrict the access of cortisol to target cells and regulate its bioavailability and metabolic clearance. On the other hand, CBG may act as a carrier protein for cortisol mediating its delivery to sites of inflammation [28,29]. CBG is also present in several tissues and may be involved in the regulation of tissue-specific GC action. For example, the significantly lower CBG level in the adipose tissue of obese Zucker rats may contribute to insulin resistance [30]. CBG levels are down regulated by physiological changes such as stress [31-33].

Both cortisol and cortisone are metabolized in liver first by the A-ring reductases followed by several steps of further structural transformation catalyzed by other enzymes [20]. The final metabolites, 5α – and 5β-tetrahydrocortisol (5α – and 5β-THF) and 5β-tetrahydrocortisone (THE), are eliminated through urinary excretion and are often used as biomarkers for GC metabolism [20,34]. While the total urinary tetrahydro metabolites (THF and THE) may serve as an indicator for GC metabolism or activity, using the ratio of the urinary THF to THE to predict the interconversion of cortisol and cortisone by 11β-HSDs is questionable for the following reasons: First, the ratio is a reflection of the total metabolism of cortisol and cortisone in the whole body instead of one particular tissue because the two isozymes have distinct tissue distribution patterns. Second, other enzymes, including the A-ring reductases and those involved in the subsequent metabolic steps forming THF and THE, also contribute to the balance between cortisol and cortisone. Therefore, the urinary ratio of THF to THE is determined by the combined activities of different enzymes in multiple tissues. Another convenient way to measure GC metabolism is to measure the salivary cortisol levels [20].

**Figure 1:**
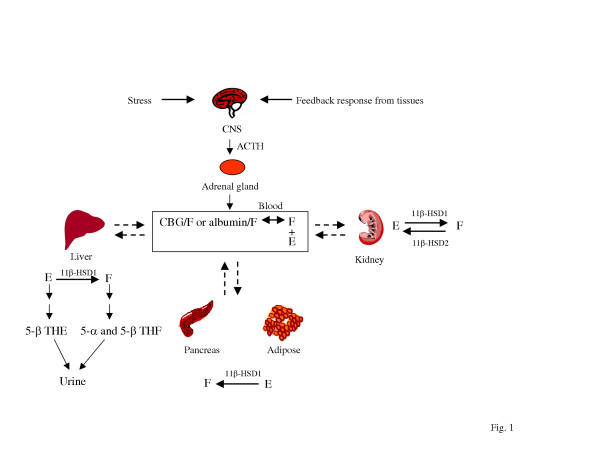
Glucocorticoid metabolism. The secretion of glucocorticoids by the adrenal gland is regulated by the HPA axis via secretion of ACTH. The main plasma cortisol (F) is protein bound with 4–5% free fraction. The plasma cortisone (E) is in the free unbound form. The equilibrium of cortisol and cortisone between the plasma and tissues are illustrated with the dotted bidirectional arrows. Tissue-specific GC metabolism are also depicted. GCs are metabolized primarily in liver and the metabolites are excreted in the urine. Only tissues relevant to the Metabolic Syndrome are shown. THE, tetrahydrocortisone; THF, tetrahydrocortisol.

GC action is mediated by GR, a nuclear receptor that regulates physiological events through activation or repression of target genes involved in inflammation, gluconeogenesis and adipocyte differentiation [35,36]. Upon activation, a GR dimer binds to GC response elements (GREs), interacts with components of the transcription machinery and activates the transcription of downstream genes [35,36]. The ligand-bound GR could also bind to negative GREs (nGREs) that mediate the repression of gene transcription, or the starting point of transcription and thus interferes with the general transcription machinery [35,36]. Some transrepression effects of GC action are achieved through a DNA binding-independent process, in which GR interacts with transcription factors such as AP-1 and NFκB and represses their activity on gene expression [37-39]. Repression of NFκB mediated transcription by GC can also be achieved by induction of IκB synthesis [40,41]. Examples of genes regulated by GR and involved in the hepatic gluconeogenesis, adipocyte differentiation, hormonal control, and inflammation are summarized in Table 1[39,42-66]. The gene stimulation or suppression effects mediated by activated GR sequentially regulate a myriad of physiological actions in response to GCs. Since the pool of active cortisol or corticosterone is the active ligand for GR, the availability of free cortisol or corticosterone mediated largely by CBG-dependent protein binding and tissue-specific activities of 11β-HSDs are critical for GC action. The role of GC action in obesity and insulin resistance is implicated by the biological or physiological consequences of deficiency or activation of CBG or 11β-HSDs (see below). The GC production and tissue-specific conversions are illustrated in Figure 1.

**Table 1:** Examples of genes regulated by GR

Gene Names	Function	Regulation	Reference
Glutamine synthetase	Amino acid metabolism	Up	42
TAT	Amino acid catabolism	Up	43, 44
Tryptophan oxygenase	Amino acid catabolism	Up	45
PEPCK (liver)	Gluconeogenesis	Up	46
G6Pase	Gluconeogenesis	Up	47, 48
Angiotensinogen	Precursor of angiotensin I; vasoconstriction, electrolyte balance, etc.	Up	49
			
Leptin	Energy metabolism	Up	50
VLDLR	Lipoprotein metabolism	Up	51
PEPCK (adipose)	Glyceroneogenesis	Down	52
aP2	Intracellular lipid shuttling and metabolism	Up	53
GLUT4	Glucose transport	Up	53
HSL	Lipolysis	Up	53
LPL	Lipid metabolism	Up	53
TNF-α	Inflammation and apoptosis	Down	53
			
Osteocalcin	Marker for mature osteoblasts	Down	54, 55
CRH	Stress mediated/feedback hormone release	Down	56
POMC	Precursor of pituitary hormones	Down	57, 58
Prolactin	Hormone critical for reproduction	Down	59
Proliferin	Angiogenesis	Down	60, 61
Glycoprotein hormone α-subunit	Common subunit of gonadotropin hormones	Down	62, 63
IL-6	Proinflammatory cytokine	Down	64
IL-8	Proinflammatory cytokine	Down	65
Collagenase	Matrix protease	Down	66
ICAM-1	Inflammatory response	Down	39

*Abbreviations*: TAT, tyrosine aminotransferase; PEPCK, phosphoenolpyruvate carboxykinase; G6Pase, glucose-6-phosphatase; VLDLR, very low density lipoprotein receptor; aP2, adipocyte fatty acid binding protein or A-FABP; GLUT-4, glucose transporter 4; HSL, hormone sensitive lipase; LPL, lipoprotein lipase; TNF-α, tumor necrosis factor α; CRH, corticotrophin-releasing hormone; POMC, proopiomelanocortin; IL-6, interleukin 6; IL-8, interleukin 8; ICAM-1, intercellular adhesion molecule 1.

## Clinical association of GC action and the Metabolic Syndrome

Accumulating clinical evidence has demonstrated the association of abnormal GC metabolism and the Metabolic Syndrome. The plasma cortisol levels were increased in an elderly cohort with one or more features of the Metabolic Syndrome [67]. Further, a good correlation was observed between total urinary GC metabolites and the number of features of the Metabolic Syndrome in these patients [67]. Both the secretion rate and peripheral clearance of cortisol in these patients were positively correlated with systolic blood pressure, fasting glucose and insulin [67]. In agreement with this finding, stress-related cortisol secretion in a population of 51-yr-old men showed associations with diastolic blood pressure, fasting glucose and insulin [68]. Several additional reports also suggest correlation of increased GC activity with insulin resistance, hyperglycemia and hypertension [69-71]. Although one study indicated that plasma cortisol levels decreased in obese women due to increased metabolic clearance [72], stress-induced cortisol response is consistently correlated with obesity in independent studies suggesting increased HPA activity in obesity [73-77]. Higher adrenocortical activity was also observed in children with higher body fat mass [78,79]. Weight loss led to lower plasma cortisol and reduced insulin resistance [79]. A study in the general population indicates that even modestly increased cortisol levels contribute to obesity [80], and insulin resistance is positively associated with cortical activity [81,82]. These clinical findings demonstrate the strong correlation of increased GC activity with the features of the Metabolic Syndrome in humans.

## The metabolic effects of GCs

The clinical correlation studies raised the possibility that GC action could play a role in the origin of the features of the Metabolic Syndrome. This notion was further established and supported by animal studies to address the metabolic effects of GCs. Adrenalectomy in young *ob/ob *or *db/db *mice slowed body weight gain [83]. Upon GC administration, these animals retained body weight gain with concomitant increase in food intake [83]. Likewise, obese Zucker rats lost body fat mass after adrenalectomy and remained so even after exogenous administration of low doses of GCs [84]. The adrenalectomy resulted in significantly reduced plasma insulin, glucose and triglyceride levels [84]. As the doses of administered GCs increased, the plasma insulin and triglyceride levels were elevated [84]. Similar results were observed in another study using adrenalectomized rats with diet-induced obesity demonstrating the effects of GC action on plasma and liver triglyceride levels, plasma insulin, and adipose tissue weight [85]. These effects appear to be minimized when there is restriction on high-energy diet [86], suggesting they may be exerted via mediating the central ingestive behavior. These findings highlight the central role of GCs in the development of obesity and other features of the Metabolic Syndrome.

**Figure 2:**
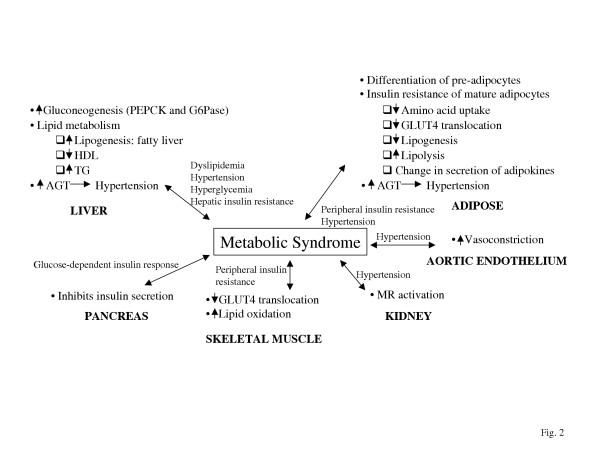
The link between the metabolic effects of glucocorticoids and the features of the Metabolic Syndrome. The major effects in different tissues are summarized and the potential physiological links to the Metabolic Syndrome are shown.

The metabolic effects of GCs are mediated by several mechanisms that are physiologically relevant to hepatic and peripheral insulin resistance, dyslipidemia, obesity and hyperglycemia. Events driven by these mechanisms take place across the tissues contributing to the abnormalities in the Metabolic Syndrome (Fig. 2). In liver, GCs increase the activities of enzymes involved in fatty acid synthesis and promote the secretion of lipoproteins [87,88]. The hepatic lipogenic effect of GCs is consistent with clinical findings that GC therapy causes triglyceride accumulation within the liver [89-91]. Since liver fat appears to be involved in the negative regulation of hepatic insulin sensitivity [92] and is associated with certain features of the Metabolic Syndrome independent of visceral fat mass [93-96], hepatic fat accumulation promoted by GCs is likely to contribute to the pathophysiology of the Metabolic Syndrome. GCs also induce the hepatic gluconeogenic pathway via the activation of GR, which stimulates the expression of phosphoenolpyruvate carboxykinase (PEPCK) and glucose-6-phosphatase (G6Pase), the rate-limiting enzymes in gluconeogenesis [97,98]. This results in increased hepatic glucose output and hyperglycemia. In adipose tissue, GCs promote the differentiation of pre-adipocytes to adipocytes, which could lead to increased body fat mass [99,100]. However, once differentiated, the adipocytes develop insulin resistance in the presence of GCs with decreased insulin-stimulated glucose uptake without changing their ability to bind insulin [101]. The reduced insulin sensitivity appears to be mediated by GC antagonizing the insulin-stimulated translocation of glucose transporters from intracellular compartments to the plasma membrane [102-104]. A similar mechanism is likely responsible for the GC-induced insulin resistance in skeletal muscle [105]. GCs also inhibit insulin-stimulated amino acid uptake by adipocytes [106]. Increased lipolysis or lipid oxidation could be also involved in the peripheral insulin resistance induced by GCs [107,108]. GCs inhibit insulin secretion by the pancreatic β cells in animals and perturb high-frequency insulin release in the fasting state in human [109,110]. GC action has been implicated in hypertension as well. GCs are agonists of mineralocorticoid receptor (MR), which upon activation leads to renal salt retention and elevated blood pressure. The expression of both 11β-HSD1 and 11β-HSD2 in kidney suggests the interconversion of inert and active GCs is maintained in a balance so that MR activation can be controlled tissue-specifically [111]. GC excess as a result of either increased 11β-HSD1 activity or reduced 11β-HSD2 activity leads to MR activation and hypertension. GCs also increase aortic vasoconstriction through unknown mechanisms. The expression of 11β-HSD1 in aortic endothelial cells is consistent with such a notion and suggests this could be a second pathway for GC induced hypertension [112-114].

These data, both physiologically and mechanistically, suggest that the metabolic effects of GCs are exerted in multiple tissues and increased GC action contributes to the etiology of the Metabolic Syndrome. Through molecular and genetic studies, more information has become available to dissect the role of tissue-specific GC action in the features of the Metabolic Syndrome. Genetic studies with the main players in GC action have been most revealing. Since GR has been well reviewed in other publications, this review will only discuss CBG and 11β-HSD1.

## Modulation of GC action by CBG is associated with adiposity

CBG is not only in the blood but also found in tissues [115,116]. Since CBG is the main GC binding protein, its tissue distribution and local levels play important roles in GC action. Intuitively, CBG level should be negatively correlated with the free cortisol or corticosterone concentration because of its role in restricting free GC fraction. This is especially true in a tissue-specific manner. For example, the reduced adipose CBG level in obese Zucker rat results in elevated free local corticosterone that may have contributed to the obesity and insulin resistance phenotype [30]. In general, in the human population, serum CBG levels are negatively correlated with a variety of parameters important in defining the Metabolic Syndrome: body mass index (BMI), waist to hip ratio (WHR), blood pressure and HOMA [117]. However, over-expression or secretion of CBG in the liver could lead to compensatory activation of the HPA axis and consequently elevated adrenal production of cortisol or corticosterone. This feedback response leads to a global effect of elevated total and free cortisol or corticosterone levels. This was observed in a pig genetic model with high fat deposits and low muscle content, where the hepatic CBG expression was significantly higher than in another population and the total and free cortisol levels were elevated [118]. On the other hand, drastic reduction of CBG concentration or its capacity to bind cortisol or corticosterone can also cause compensatory response by the HPA axis. A familial CBG deficiency led to decreased total and free plasma cortisol levels and hypotension [119]. Likewise, a human CBG polymorphism associated with reduced affinity for cortisol only led to a marginal increase in serum free cortisol, possibly due to the negative regulation of cortisol production by the HPA axis [120]. Together, these data demonstrate the importance of CBG level and its cortisol binding capacity in modulating GC action and origination of the Metabolic Syndrome. Further, these studies also suggest that the variation in CBG level or capacity may trigger compensatory response of the HPA axis to balance plasma free cortisol concentrations.

Despite the compensatory response by the HPA axis to balance the plasma free cortisol or corticosterone concentrations under conditions of CBG reduction or deficiency, tissue-specific GC excess can still occur. This is especially important with respect to GC-stimulated differentiation of pre-adipocytes and insulin resistance of mature adipocytes, with the former effect increasing fat content and the latter reducing the tissue sensitivity to insulin. For instance, preadipocytes from an individual with CBG deficiency had increased proliferation and enhanced differentiation compared to normal cells [121], which may be responsible for the increased adiposity in CBG deficiency. This notion was observed in genetic models of obesity and insulin resistance. The CBG capacity the white adipose tissue of Zucker rat is lower than that in its lean counterpart [30,122], suggesting increased GC action in the obese adipose tissue that could contribute to the obese and insulin resistance phenotype.

## 11β-HSD1 and obesity and insulin resistance

Both 11β-HSD1 and 11β-HSD2 are located at the endoplasmic reticulum (ER) but with distinct topologies. 11β-HSD1 has one short N-terminal transmembrane region with the catalytic domain protruding into the ER lumen; in contrast, the N-terminus of 11β-HSD2 is lumenal with the catalytic domain facing the cytoplasm [123-125]. The primary role of 11β-HSD2 is to prevent renal GC excess and consequent MR activation by inactivating cortisol or corticosterone, as mice deficient in 11β-HSD2 had hypokalemia and hypertension [126]. Given the growing interest in 11β-HSD1 and its role in the Metabolic Syndrome, this section will primarily focus on this isozyme.

Dysregulation of tissue-specific 11β-HSD1 expression and activity has been observed in obese diabetic animal models and humans. Compared with their lean littermates, *ob/ob *mice have reduced hepatic 11β-HSD1 activity but higher corticosterone level in liver due to their elevated plasma corticosterone [127]. As a result, the liver PEPCK expression is elevated at least partly contributing to hyperglycemia. However, the hepatic 11β-HSD1 activity is marginally increased in *db/db *mice [128]. As in *ob/ob *mice, the 11β-HSD1 activity is decreased in liver but increased in omental fat in obese Zucker rats [129,130]. Although both impaired hepatic regeneration of cortisol by 11β-HSD1 and elevated adipose 11β-HSD1 activity were observed in obese humans [131,132], the association of adipose 11β-HSD1 activity with obesity, insulin resistance and other features of the Metabolic Syndrome has been consistently observed in different groups of obese subjects, including obese men and women [131,133,134]. However, no difference in 11β-HSD1 activity was detected between obese type 2 diabetics and their obese controls, suggesting the dysregulation of 11β-HSD1 is better associated with obesity than the diabetic phenotype [135]. In-situ hybridization revealed that 11β-HSD1 mRNA is increased in both subcutaneous and visceral fat in obese subjects [136]. The association of adipose 11β-HSD1 with BMI and other features of the Metabolic Syndrome was also found in populations of different ethnic backgrounds [137]. In a group of young adult monozygotic twins, the intrapair differences in BMI are positively correlated with those in adipose 11β-HSD1 expression [138]. This association is clearly established on the same genetic background, confirming the direct link of adipose 11β-HSD1 activity and adiposity. Most of these association studies were done with subcutaneous fat. It is important to note that 11β-HSD1 activity is higher in omental fat and subject to stimulation [139]. The activity of 11β-HSD1 in adipocytes is relevant for the correlation since the activity in cultured preadipocytes does not appear to be correlated with obesity [140]. These association studies suggest that the adipose 11β-HSD1 may be a contributing factor to obesity and insulin resistance. In agreement with this conclusion, treatment of obese Zucker rats with carbenoxolone slightly improved lipid profile but had no effect on obesity and insulin resistance, because only the hepatic 11β-HSD1 but not that in adipose tissue was inhibited [141]. It is important to note that carbenoxolone also inhibits 11β-HSD2 and further studies with selective 11β-HSD1 inhibitors are needed to confirm this observation. In contrast to increased adiposity in the Metabolic Syndrome, some human immunodeficiency virus (HIV)-infected patients treated with combined highly active antiretroviral therapy (HAART) develop a lipodystrophic syndrome. The condition is characterized with loss of subcutaneous fat, accumulation of abdominal fat, hypertriglyceridemia and insulin resistance [142]. The condition is also referred to as pseudo-Cushing's syndrome because the distribution of fat accumulation in these patients is similar to that in Cushing's syndrome but their circulating cortisol levels are not elevated [143]. Interestingly, patients with lipodystrophy were shown to have higher levels of subcutaneous adipose 11β-HSD1 expression and higher ratios of urinary cortisol:cortisone metabolites than non-lipodystrophic patients [144]. These findings suggest that 11β-HSD1 could play a role in mediating the metabolic abnormalities of the HAART-associated lipodystrophy with the almost complete loss of subcutaneous fat. This further suggests that the expression of 11β-HSD1 seems to be more important to the metabolic state than the amount of subcutaneous fat though further investigation is required.

Genetic studies using animal models support the findings in the clinical studies. In mice deficient in 11β-HSD1 generated through targeted gene disruption, there was no conversion of the inert 11-dehydrocorticosterone to corticosterone and attenuation of the hepatic activities of PEPCK and G6Pase, two key gluconeogenic enzymes [145]. These mice consumed more calories but were resistant to high fat diet-induced obesity, insulin resistance and hyperglycemia with improved lipoprotein profile [145-147]. Concomitant with these phenotypes, the adipose expression of TNF-α decreased, and adiponectin, PPARγ, and UCP-2 increased indicating insulin sensitization [146]. There were no bone marrow adipocytes in these knockout animals but bone formation appeared to be normal, suggesting that intracellular GC action may not play a role in bone formation [148]. The HPA axis appears to be activated in the 11β-HSD1 knockout mice. There was compensatory adrenal hyperplasia, increased secretion of corticosterone and exaggerated ACTH and corticosterone response to stress [145,149]. The plasma CBG levels were slightly reduced [149]. These findings with 11β-HSD1 deficiency suggest inhibition of this enzyme could help ameliorate some of the features of the Metabolic Syndrome. However, compensatory response from the HPA axis and the induced adrenal activity can occur. Interestingly, 11β-HSD1 knockout ameliorated age-related learning impairments but the underlying mechanism is not clear [150]. The importance of 11β-HSD1 in the Metabolic Syndrome was also demonstrated with 11β-HSD1 transgenic animals. Mice with adipose-specific overexpression of the rat 11β-HSD1 had increased adipose levels of corticosterone and acquired features of the Metabolic Syndrome: diet-induced visceral obesity, insulin resistance, hyperlipidemia and hyperphagia [151]. The transgenic mice also developed hypertension, at least in part due to the increased adipose expression of angiotensinogen and the consequent activation of the rennin-angiotensin system (RAS) [152]. In contrast, selective overexpression of 11β-HSD1 in liver only caused mild insulin resistance with no effect on fat depot mass [153], although impaired hepatic lipid clearance and hypertension were observed in these animals. These transgenic studies demonstrate that both the hepatic and adipose 11β-HSD1 activities contribute in some way to insulin resistance and other features of the Metabolic Syndrome. However, the adipose activity appears to be correlated with a stronger phenotype of obesity and insulin resistance and therefore is likely the primary target for the treatment of insulin resistance. The hepatic 11β-HSD1 activity, although secondary, appears to be more important in improving lipid metabolism and controlling blood pressure. Several cases of human 11β-HSD1 deficiency have been reported. The ability of these subjects to convert cortisone to cortisol upon dexamethasone suppression was apparently compromised [154-158]. These patients appeared to be normal except for mild adrenal hyperplasia in some cases, and hirsutism, and elevated plasma cortisol levels [154-158]. Unfortunately, insufficient insulin sensitivity data have been reported with these patients. Although both obese and lean patients with 11β-HSD1 deficiency have been identified, it is not clear if the body weight is associated with 11β-HSD1 deficiency. However, polymorphisms in the 11β-HSD1 gene have been linked to adiposity in association studies with human subjects [159,160].

## Inhibitors of GC action

Given its important role in the Metabolic Syndrome, antagonizing GC action has been taken as an approach to treat some features of the Metabolic Syndrome. Targeting GR is a direct approach to antagonize the GC action. The global effect on GC action by this approach could lead to the activation of the HPA axis as well as blocking the anti-inflammatory function of GCs. Inhibition of 11β-HSD1 activity offers more tissue specificity due to the limited expression pattern of this enzyme. Inhibitors for both 11β-HSD1 and GR include naturally occurring and pharmaceutically developed compounds.

The expected effects of 11β-HSD1 inhibition include reduced hepatic PEPCK and G6Pase expression to reduce hepatic glucose output; reduced adiposity and improved peripheral insulin sensitivity. Since 11β-HSD1 mediated GC action inhibits glucose-dependent insulin secretion [161] and the expression of 11β-HSD1 is significantly increased in diabetic islets [162], 11β-HSD1 inhibitors can potentially help reduce postprandial glucose excursion. Several inhibitors of 11β-HSD1 were described in the literature prior to the pharmaceutical targeting of this enzyme in recent years but none of them is selective and highly potent. Metyrapone, often used in the diagnosis of adrenal corticoid-related disease such as Cushing' syndrome, is a weak competitive inhibitor of 11β-HSD1 [163]. Other inhibitors include liquorice derivatives carbenoxolone (CBX) and glycyrrhetinic acid (GE) [164]. GE is more potent against the dehydrogenase activity and CBX is almost equally potent against activities of both directions (dehydrogenase and reductase). Although far more potent than other inhibitors, CBX and GE are not selective because they also inhibit 11β-HSD2. Chenodeoxycholic acid (CDCA) inhibits 11β-HSD1 with a potency of micromolar range but studies of its activity against 11β-HSD2 have generated conflicting results [165-167]. Although not selective, CBX has been used in human studies where it reduced glucose production during hyperglucagonemia largely due to its suppressive effect on glycogenolysis in lean male patients with type 2 diabetes [168]. Interestingly, CBX also improved verbal frequency and memory in healthy elderly men and patients with type 2 diabetes [169]. This is consistent with findings in 11β-HSD1 knockout mice [150]. Selective 11β-HSD1 inhibitors have been developed for pharmaceutical use in recent years. These inhibitors have been shown to be efficacious in diabetic animal models [170-173].

GR antagonists were developed on the rationale that activated GR stimulates PEPCK and G6Pase, the two key enzymes in hepatic gluconeogenesis that increases the hepatic glucose output [97,98,174]. Since hepatic gluconeogenesis in diabetics is increased [175], inhibition of hepatic GR action is expected for glucose lowering in diabetics. A well-known GR antagonist is RU-486, which was also found to have agonist activities [176]. Although efficacious [177], long-term systemic treatment with a GR antagonist may activate the HPA axis and increases cortisol secretion [178]. Other GR antagonists were also reported but without resolving the issue of HPA activation [179]. Selective inhibition of the hepatic GR activation in a non-systemic manner could provide advantages with no undesirable side effects. Liver selective targeting of the drug appears to be a good strategy [180].

## Conclusions

GCs are stress hormones with a wide spectrum of physiological effects and have been implicated in the pathophysiology of the Metabolic Syndrome. This notion has been supported by animal studies and clinical findings. The GC action appears to mediate certain aspects of the Metabolic Syndrome. In that regard, targeting key players in the GC action is expected to be a viable approach to treat some or all the features of the Metabolic Syndrome. However, cautions should be taken because the GC metabolism is regulated by the HPA axis and inhibition of GC pathways could lead to the activation of HPA axis and elevated adrenal cortisol secretion. To avoid the compensatory feedback response, efforts to separate the effect of GC modulators from HPA activity is needed. Although challenging, this could be achieved by tissue-specific modulation of GC action by targeting drugs to tissues of interest while sparing others, especially the CNS where HPA activation occurs. The availability of small molecule compounds will facilitate this type of studies in animal models to further dissect the regulatory function of the HPA axis and help assess whether tissue selective modulation of GC action without triggering the HPA axis is achievable.

## Declaration of competing interests

The author is an employed researcher in a biopharmaceutical company.

## References

[B1] Reaven GM (1988). Role of insulin resistance in human disease. Banting lecture. Diabetes.

[B2] Reaven GM (1991). Insulin resistance, hyperinsulinemia, hypertriglyceridemia, and hypertension. Parallels between human disease and rodent models. Diabetes Care.

[B3] DeFronzo RA, Ferrannini E (1991). Insulin resistance. A multifaceted syndrome responsible for NIDDM, obesity, hypertension, dyslipidemia, and atherosclerotic cardiovascular disease. Diabetes Care.

[B4] (2002). Third Report of the National Cholesterol Education Program (NCEP) Expert Panel on Detection, Evaluation, and Treatment of High Blood Cholesterol in Adults (Adult Treatment Panel III). Final Report. Circulation.

[B5] World Health Organization (1999). Definition, diagnosis and classification of diabetes mellitus and its complications: report of a WHO Consultation. Part 1: diagnosis and classification of diabetes mellitus. Geneva, Switzerland: World Health Organization. http://whqlibdoc.who.int/hq/1999/WHO_NCD_NCS_99.2.pdf.

[B6] Alberti KG, Zimmet PZ (1998). Definition, diagnosis and classification of diabetes mellitus and its complications. Part 1: diagnosis and classification of diabetes mellitus provisional report of a WHO consultation. Diabet Med.

[B7] Grundy SM, Brewer HB, Cleeman JI, Smith SC, Lenfant C, For the Conference participants (2004). Definition of metabolic syndrome: Report of the National Heart, Lung, and Blood Institute/American Heart Association conference on scientific issues related to definition. Circulation.

[B8] Ford ES, Giles WH, Dietz WH (2002). Prevalence of the metabolic syndrome among US adults: findings from the third National Health and Nutrition Examination Survey. JAMA.

[B9] Trevisan M, Liu J, Bahsas FB, Menotti A (1998). Syndrome X and mortality: a population-based study. Risk Factor and Life Expectancy Research Group. Am J Epidemiol.

[B10] Isomaa B, Almgren P, Tuomi T, Forsen B, Lahti K, Nissen M, Taskinen MR, Groop L (2001). Cardiovascular morbidity and mortality associated with the metabolic syndrome. Diabetes Care.

[B11] Arnaldi G, Angeli A, Atkinson AB, Bertagna X, Cavagnini F, Chrousos GP, Fava GA, Findling JW, Gaillard RC, Grossman AB, Kola B, Lacroix A, Mancini T, Mantero F, Newell-Price J, Nieman LK, Sonino N, Vance ML, Giustina A, Boscaro M (2003). Diagnosis and complications of Cushing's syndrome: a consensus statement. J Clin Endocrinol Metab.

[B12] Magiakou MA, Mastorakos G, Zachman K, Chrousos GP (1997). Blood pressure in children and adolescents with Cushing's syndrome before and after surgical cure. J Clin Endocrinol Metab.

[B13] Fallo F, Sonino N, Barzon L, Pistorello M, Pagotto U, Paoletta A, Boscaro M (1996). Effect of surgical treatment on hypertension in Cushing's syndrome. Am J Hypertens.

[B14] Davis GF (1986). Adverse effects of corticosteroids: II. Systemic. Clin Dermatol.

[B15] Gallant C, Kenny P (1986). Oral glucocorticoids and their complications. J Am Acad Dermatol.

[B16] Covar RA, Leung DY, McCormick D, Steelman J, Zeitler P, Spahn JD (2000). Risk factors associated with glucocorticoid-induced adverse effects in children with severe asthma. J Allergy Clin Immunol.

[B17] Srivastava LS, Werk EE, Thrasher K, Sholiton LJ, Kozera R, Nolten W, Knowles HC (1973). Plasma cortisone concentration as measured by radioimmunoassay. J Clin Endocrinol Metab.

[B18] Reach G, Nakane H, Nakane Y, Auzan C, Corvol P (1977). Cortisol metabolism and excretion in the isolated perfused rat kidney. Steroids.

[B19] Whitworth JA, Stewart PM, Burt D, Atherden SM, Edwards CR (1989). The kidney is the major site of cortisone production in man. Clin Endocrinol (Oxf).

[B20] Walker EA, Stewart PM (2003). 11β-hydroxysteroid dehydrogenase: unexpected connections. Trends Endocrinol Metab.

[B21] Bujalska I, Shimojo M, Howie A, Stewart PM (1997). Human 11β-hydroxysteroid dehydrogenase: studies on the stably transfected isoforms and localization of the type 2 isozyme within renal tissue. Steroids.

[B22] Walker BR, Campbell JC, Fraser R, Stewart PM, Edwards CR (1992). Mineralocorticoid excess and inhibition of 11β-hydroxysteroid dehydrogenase in patients with ectopic ACTH syndrome. Clin Endocrinol (Oxf).

[B23] Johansson A, Andrew R, Forsberg H, Cederquist K, Walker BR, Olsson T (2001). Glucocorticoid metabolism and adrenocortical reactivity to ACTH in myotonic dystrophy. J Clin Endocrinol Metab.

[B24] Lu XY, Shieh KR, Kabbaj M, Barsh GS, Akil H, Watson SJ (2002). Diurnal rhythm of agouti-related protein and its relation to corticosterone and food intake. Endocrinology.

[B25] Delbende C, Delarue C, Lefebvre H, Bunel DT, Szafarczyk A, Mocaer E, Kamoun A, Jegou S, Vaudry H (1992). Glucocorticoids, transmitters and stress. Br J Psychiatry Suppl.

[B26] Dunn JF, Nisula BC, Rodbard D (1981). Transport of steroid hormones: binding of 21 endogenous steroids to both testosterone-binding globulin and corticosteroid-binding globulin in human plasma. J Clin Endocrinol Metab.

[B27] Weiser JN, Do YS, Feldman D (1979). Synthesis and secretion of corticosteroid-binding globulin by rat liver. A source of heterogeneity of hepatic corticosteroid-binders. J Clin Invest.

[B28] Pemberton PA, Stein PE, Pepys MB, Potter JM, Carrell RW (1988). Hormone binding globulins undergo serpin conformational change in inflammation. Nature.

[B29] Hammond GL, Smith CL, Paterson NA, Sibbald WJ (1990). A role for corticosteroid-binding globulin in delivery of cortisol to activated neutrophils. J Clin Endocrinol Metab.

[B30] Grasa MM, Cabot C, Balada F, Virgili J, Sanchis D, Monserrat C, Fernandez-Lopez JA, Remesar X, Alemany M (1998). Corticosterone binding to tissues of adrenalectomized lean and obese Zucker rats. Horm Metab Res.

[B31] Vogeser M, Felbinger TW, Kilger E, Roll W, Fraunberger P, Jacob K (1999). Corticosteroid-binding globulin and free cortisol in the early postoperative period after cardiac surgery. Clin Biochem.

[B32] Bernier J, Jobin N, Emptoz-Bonneton A, Pugeat MM, Garrel DR (1998). Decreased corticosteroid-binding globulin in burn patients: relationship with interleukin-6 and fat in nutritional support. Crit Care Med.

[B33] Tinnikov AA, Legan MV, Sheveluk NA, Cvetovskaya GA, Naumenko SE, Sidelnikov SG (1996). Corticosteroid and immune responses to cardiac surgery. Steroids.

[B34] Aranoff G, Rosler A (1980). Urinary tetrahydrocortisone and tetrahydrocortisol glucosiduronates in normal newborns, children and adults. Acta Endocrinol (Copenh).

[B35] Bamberger CM, Schulte HM, Chrousos GP (1996). Molecular determinants of glucocorticoid receptor function and tissue sensitivity to glucocorticoids. Endocr Rev.

[B36] Schacke H, Rehwinkel H (2004). Dissociated glucocorticoid receptor ligands. Curr Opin Investig Drugs.

[B37] Zhang XK, Dong JM, Chiu JF (1991). Regulation of alpha-fetoprotein gene expression by antagonism between AP-1 and the glucocorticoid receptor at their overlapping binding site. J Biol Chem.

[B38] Ray A, Prefontaine KE (1994). Physical association and functional antagonism between the p65 subunit of transcription factor NF-kappa B and the glucocorticoid receptor. Proc Natl Acad Sci U S A.

[B39] Nissen RM, Yamamoto KR (2000). The glucocorticoid receptor inhibits NFkappaB by interfering with serine-2 phosphorylation of the RNA polymerase II carboxy-terminal domain. Genes Dev.

[B40] Scheinman RI, Cogswell PC, Lofquist AK, Baldwin AS (1995). Role of transcriptional activation of I kappa B alpha in mediation of immunosuppression by glucocorticoids. Science.

[B41] Auphan N, DiDonato JA, Rosette C, Helmberg A, Karin M (1995). Immunosuppression by glucocorticoids: inhibition of NF-kappa B activity through induction of I kappa B synthesis. Science.

[B42] Gaunitz F, Heise K, Schumann R, Gebhardt R (2002). Glucocorticoid induced expression of glutamine synthetase in hepatoma cells. Biochem Biophys Res Commun.

[B43] Becker PB, Gloss B, Schmid W, Strahle U, Schutz G (1986). In vivo protein-DNA interactions in a glucocorticoid response element require the presence of the hormone. Nature.

[B44] Jantzen HM, Strahle U, Gloss B, Stewart F, Schmid W, Boshart M, Miksicek R, Schutz G (1987). Cooperativity of glucocorticoid response elements located far upstream of the tyrosine aminotransferase gene. Cell.

[B45] Danesch U, Gloss B, Schmid W, Schutz G, Schule R, Renkawitz R (1987). Glucocorticoid induction of the rat tryptophan oxygenase gene is mediated by two widely separated glucocorticoid-responsive elements. EMBO J.

[B46] Imai E, Stromstedt PE, Quinn PG, Carlstedt-Duke J, Gustafsson JA, Granner DK (1990). Characterization of a complex glucocorticoid response unit in the phosphoenolpyruvate carboxykinase gene. Mol Cell Biol.

[B47] Schmoll D, Allan BB, Burchell A (1996). Cloning and sequencing of the 5' region of the human glucose-6-phosphatase gene: transcriptional regulation by cAMP, insulin and glucocorticoids in H4IIE hepatoma cells. FEBS Lett.

[B48] Lin B, Morris DW, Chou JY (1998). Hepatocyte nuclear factor 1alpha is an accessory factor required for activation of glucose-6-phosphatase gene transcription by glucocorticoids. DNA Cell Biol.

[B49] Kalinyak JE, Perlman AJ (1987). Tissue-specific regulation of angiotensinogen mRNA accumulation by dexamethasone. J Biol Chem.

[B50] Slieker LJ, Sloop KW, Surface PL, Kriauciunas A, LaQuier F, Manetta J, Bue-Valleskey J, Stephens TW (1996). Regulation of expression of ob mRNA and protein by glucocorticoids and cAMP. J Biol Chem.

[B51] Ensler K, Mohammadieh M, Broijersen A, Angelin B, Gafvels M (2002). Dexamethasone stimulates very low density lipoprotein (VLDL) receptor gene expression in differentiating 3T3-L1 cells. Biochim Biophys Acta.

[B52] Olswang Y, Blum B, Cassuto H, Cohen H, Biberman Y, Hanson RW, Reshef L (2003). Glucocorticoids repress transcription of phosphoenolpyruvate carboxykinase (GTP) gene in adipocytes by inhibiting its C/EBP-mediated activation. J Biol Chem.

[B53] Zilberfarb V, Siquier K, Strosberg AD, Issad T (2001). Effect of dexamethasone on adipocyte differentiation markers and tumour necrosis factor-alpha expression in human PAZ6 cells. Diabetologia.

[B54] Stromstedt PE, Poellinger L, Gustafsson JA, Carlstedt-Duke J (1991). The glucocorticoid receptor binds to a sequence overlapping the TATA box of the human osteocalcin promoter: a potential mechanism for negative regulation. Mol Cell Biol.

[B55] Meyer T, Carlstedt-Duke J, Starr DB (1997). A weak TATA box is a prerequisite for glucocorticoid-dependent repression of the osteocalcin gene. J Biol Chem.

[B56] Malkoski SP, Dorin RI (1999). Composite glucocorticoid regulation at a functionally defined negative glucocorticoid response element of the human corticotropin-releasing hormone gene. Mol Endocrinol.

[B57] Charron J, Drouin J (1986). Glucocorticoid inhibition of transcription from episomal proopiomelanocortin gene promoter. Proc Natl Acad Sci U S A.

[B58] Drouin J, Sun YL, Chamberland M, Gauthier Y, De Lean A, Nemer M, Schmidt TJ (1993). Novel glucocorticoid receptor complex with DNA element of the hormone-repressed POMC gene. EMBO J.

[B59] Sakai DD, Helms S, Carlstedt-Duke J, Gustafsson JA, Rottman FM, Yamamoto KR (1988). Hormone-mediated repression: a negative glucocorticoid response element from the bovine prolactin gene. Genes Dev.

[B60] Diamond MI, Miner JN, Yoshinaga SK, Yamamoto KR (1990). Transcription factor interactions: selectors of positive or negative regulation from a single DNA element. Science.

[B61] Mordacq JC, Linzer DI (1989). Co-localization of elements required for phorbol ester stimulation and glucocorticoid repression of proliferin gene expression. Genes Dev.

[B62] Akerblom IE, Slater EP, Beato M, Baxter JD, Mellon PL (1988). Negative regulation by glucocorticoids through interference with a cAMP responsive enhancer. Science.

[B63] Chatterjee VK, Madison LD, Mayo S, Jameson JL (1991). Repression of the human glycoprotein hormone alpha-subunit gene by glucocorticoids: evidence for receptor interactions with limiting transcriptional activators. Mol Endocrinol.

[B64] Waage A, Slupphaug G, Shalaby R (1990). Glucocorticoids inhibit the production of IL6 from monocytes, endothelial cells and fibroblasts. Eur J Immunol.

[B65] Mukaida N, Morita M, Ishikawa Y, Rice N, Okamoto S, Kasahara T, Matsushima K (1994). Novel mechanism of glucocorticoid-mediated gene repression. Nuclear factor-kappa B is target for glucocorticoid-mediated interleukin 8 gene repression. J Biol Chem.

[B66] Yang-Yen HF, Chambard JC, Sun YL, Smeal T, Schmidt TJ, Drouin J, Karin M (1990). Transcriptional interference between c-Jun and the glucocorticoid receptor: mutual inhibition of DNA binding due to direct protein-protein interaction. Cell.

[B67] Andrew R, Gale CR, Walker BR, Seckl JR, Martyn CN (2002). Glucocorticoid metabolism and the Metabolic Syndrome: associations in an elderly cohort. Exp Clin Endocrinol Diabetes.

[B68] Rosmond R, Dallman MF, Bjorntorp P (1998). Stress-related cortisol secretion in men: relationships with abdominal obesity and endocrine, metabolic and hemodynamic abnormalities. J Clin Endocrinol Metab.

[B69] Walker BR, Phillips DI, Noon JP, Panarelli M, Andrew R, Edwards HV, Holton DW, Seckl JR, Webb DJ, Watt GC (1998). Increased glucocorticoid activity in men with cardiovascular risk factors. Hypertension.

[B70] Reynolds RM, Walker BR, Syddall HE, Andrew R, Wood PJ, Whorwood CB, Phillips DI (2001). Altered control of cortisol secretion in adult men with low birth weight and cardiovascular risk factors. J Clin Endocrinol Metab.

[B71] Filipovsky J, Ducimetiere P, Eschwege E, Richard JL, Rosselin G, Claude JR (1996). The relationship of blood pressure with glucose, insulin, heart rate, free fatty acids and plasma cortisol levels according to degree of obesity in middle-aged men. J Hypertens.

[B72] Strain GW, Zumoff B, Kream J, Strain JJ, Levin J, Fukushima D (1982). Sex difference in the influence of obesity on the 24 hr mean plasma concentration of cortisol. Metabolism.

[B73] Moyer AE, Rodin J, Grilo CM, Cummings N, Larson LM, Rebuffe-Scrive M (1994). Stress-induced Cortisol response and fat distribution in women. Obes Res.

[B74] Epel EE, Moyer AE, Martin CD, Macary S, Cummings N, Rodin J, Rebuffe-Scrive M (1999). Stress-induced cortisol, mood, and fat distribution in men. Obes Res.

[B75] Ljung T, Andersson B, Bengtsson BA, Bjorntorp P, Marin P (1996). Inhibition of cortisol secretion by dexamethasone in relation to body fat distribution: a dose-response study. Obes Res.

[B76] Marin P, Darin N, Amemiya T, Andersson B, Jern S, Bjorntorp P (1992). Cortisol secretion in relation to body fat distribution in obese premenopausal women. Metabolism.

[B77] Pasquali R, Cantobelli S, Casimirri F, Capelli M, Bortoluzzi L, Flamia R, Labate AM, Barbara L (1993). The hypothalamic-pituitary-adrenal axis in obese women with different patterns of body fat distribution. J Clin Endocrinol Metab.

[B78] Dimitriou T, Maser-Gluth C, Remer T (2003). Adrenocortical activity in healthy children is associated with fat mass. Am J Clin Nutr.

[B79] Reinehr T, Andler W (2004). Cortisol and its relation to insulin resistance before and after weight loss in obese children. Horm Res.

[B80] Fraser R, Ingram MC, Anderson NH, Morrison C, Davies E, Connell JM (1999). Cortisol effects on body mass, blood pressure, and cholesterol in the general population. Hypertension.

[B81] Reynolds RM, Walker BR, Syddall HE, Whorwood CB, Wood PJ, Phillips DI (2001). Elevated plasma cortisol in glucose-intolerant men: differences in responses to glucose and habituation to venepuncture. J Clin Endocrinol Metab.

[B82] Stolk RP, Lamberts SW, de Jong FH, Pols HA, Grobbee DE (1996). Gender differences in the associations between cortisol and insulin in healthy subjects. J Endocrinol.

[B83] Shimomura Y, Bray GA, Lee M (1987). Adrenalectomy and steroid treatment in obese (*ob/ob*) and diabetic (*db/db*) mice. Horm Metab Res.

[B84] Freedman MR, Horwitz BA, Stern JS (1986). Effect of adrenalectomy and glucocorticoid replacement on development of obesity. Am J Physiol.

[B85] Mantha L, Palacios E, Deshaies Y (1999). Modulation of triglyceride metabolism by glucocorticoids in diet-induced obesity. Am J Physiol.

[B86] Mantha L, Deshaies Y (2000). Energy intake-independent modulation of triglyceride metabolism by glucocorticoids in the rat. Am J Physiol Regul Integr Comp Physiol.

[B87] Diamant S, Shafrir E (1975). Modulation of the activity of insulin-dependent enzymes of lipogenesis by glucocorticoids. Eur J Biochem.

[B88] Wang CN, McLeod RS, Yao Z, Brindley DN (1995). Effects of dexamethasone on the synthesis, degradation, and secretion of apolipoprotein B in cultured rat hepatocytes. Arterioscler Thromb Vasc Biol.

[B89] Itoh S, Igarashi M, Tsukada Y, Ichinoe A (1977). Nonalcoholic fatty liver with alcoholic hyalin after long-term glucocorticoid therapy. Acta Hepatogastroenterol (Stuttg).

[B90] Nanki T, Koike R, Miyasaka N (1999). Subacute severe steatohepatitis during prednisolone therapy for systemic lupus erythematosis. Am J Gastroenterol.

[B91] Dourakis SP, Sevastianos VA, Kaliopi P (2002). Acute severe steatohepatitis related to prednisolone therapy. Am J Gastroenterol.

[B92] Samuel VT, Liu ZX, Qu X, Elder BD, Bilz S, Befroy D, Romanelli AJ, Shulman GI (2004). Mechanism of hepatic insulin resistance in non-alcoholic fatty liver disease. J Biol Chem.

[B93] Seppala-Lindroos A, Vehkavaara S, Hakkinen AM, Goto T, Westerbacka J, Sovijarvi A, Halavaara J, Yki-Jarvinen H (2002). Fat accumulation in the liver is associated with defects in insulin suppression of glucose production and serum free fatty acids independent of obesity in normal men. J Clin Endocrinol Metab.

[B94] Marchesini G, Brizi M, Morselli-Labate AM, Bianchi G, Bugianesi E, McCullough AJ, Forlani G, Melchionda N (1999). Association of nonalcoholic fatty liver disease with insulin resistance. Am J Med.

[B95] Tiikkainen M, Tamminen M, Hakkinen AM, Bergholm R, Vehkavaara S, Halavaara J, Teramo K, Rissanen A, Yki-Jarvinen H (2002). Liver-fat accumulation and insulin resistance in obese women with previous gestational diabetes. Obes Res.

[B96] Nguyen-Duy TB, Nichaman MZ, Church TS, Blair SN, Ross R (2003). Visceral fat and liver fat are independent predictors of metabolic risk factors in men. Am J Physiol Endocrinol Metab.

[B97] Friedman JE, Yun JS, Patel YM, McGrane MM, Hanson RW (1993). Glucocorticoids regulate the induction of phosphoenolpyruvate carboxykinase (GTP) gene transcription during diabetes. J Biol Chem.

[B98] Argaud D, Zhang Q, Pan W, Maitra S, Pilkis SJ, Lange AJ (1996). Regulation of rat liver glucose-6-phosphatase gene expression in different nutritional and hormonal states: gene structure and 5'-flanking sequence. Diabetes.

[B99] Hauner H, Schmid P, Pfeiffer EF (1987). Glucocorticoids and insulin promote the differentiation of human adipocyte precursor cells into fat cells. J Clin Endocrinol Metab.

[B100] Hauner H, Entenmann G, Wabitsch M, Gaillard D, Ailhaud G, Negrel R, Pfeiffer EF (1989). Promoting effect of glucocorticoids on the differentiation of human adipocyte precursor cells cultured in a chemically defined medium. J Clin Invest.

[B101] Olefsky JM (1975). Effect of dexamethasone on insulin binding, glucose transport, and glucose oxidation of isolated rat adipocytes. J Clin Invest.

[B102] Carter-Su C, Okamoto K (1987). Effect of insulin and glucocorticoids on glucose transporters in rat adipocytes. Am J Physiol.

[B103] Horner HC, Munck A, Lienhard GE (1987). Dexamethasone causes translocation of glucose transporters from the plasma membrane to an intracellular site in human fibroblasts. J Biol Chem.

[B104] Oda N, Nakai A, Mokuno T, Sawai Y, Nishida Y, Mano T, Asano K, Itoh Y, Kotake M, Kato S, Masunaga R, Iwase K, Tsujimura T, Itoh M, Kawabe T, Nagasaka A (1995). Dexamethasone-induced changes in glucose transporter 4 in rat heart muscle, skeletal muscle and adipocytes. Eur J Endocrinol.

[B105] Dimitriadis G, Leighton B, Parry-Billings M, Sasson S, Young M, Krause U, Bevan S, Piva T, Wegener G, Newsholme EA (1997). Effects of glucocorticoid excess on the sensitivity of glucose transport and metabolism to insulin in rat skeletal muscle. Biochem J.

[B106] Grunfeld C, Jones DS (1986). Glucocorticoid-induced insulin resistance in vitro: inhibition of insulin-stimulated methylaminoisobutyric acid uptake. Horm Metab Res.

[B107] Ekstrand A, Saloranta C, Ahonen J, Gronhagen-Riska C, Groop LC (1992). Reversal of steroid-induced insulin resistance by a nicotinic-acid derivative in man. Metabolism.

[B108] Guillaume-Gentil C, Assimacopoulos-Jeannet F, Jeanrenaud B (1993). Involvement of non-esterified fatty acid oxidation in glucocorticoid-induced peripheral insulin resistance *in vivo *in rats. Diabetologia.

[B109] Lambillotte C, Gilon P, Henquin JC (1997). Direct glucocorticoid inhibition of insulin secretion. An in vitro study of dexamethasone effects in mouse islets. J Clin Invest.

[B110] Hollingdal M, Juhl CB, Dall R, Sturis J, Veldhuis JD, Schmitz O, Porksen N (2002). Glucocorticoid induced insulin resistance impairs basal but not glucose entrained high-frequency insulin pulsatility in humans. Diabetologia.

[B111] Lloyd-MacGilp SA, Nelson SM, Florin M, Lo M, McKinnell J, Sassard J, Kenyon CJ (1999). 11β-hydroxysteroid dehydrogenase and corticosteroid action in lyon hypertensive rats. Hypertension.

[B112] Brem AS, Bina RB, King TC, Morris DJ (1998). Localization of 2 11β-OH steroid dehydrogenase isoforms in aortic endothelial cells. Hypertension.

[B113] Brem AS, Bina RB, Hill N, Alia C, Morris DJ (1997). Effects of licorice derivatives on vascular smooth muscle function. Life Sci.

[B114] Walker BR, Sang KS, Williams BC, Edwards CR (1994). Direct and indirect effects of carbenoxolone on responses to glucocorticoids and noradrenaline in rat aorta. J Hypertens.

[B115] Kuhn RW, Green AL, Raymoure WJ, Siiteri PK (1986). Immunocytochemical localization of corticosteroid-binding globulin in rat tissues. J Endocrinol.

[B116] Werthamer S, Samuels AJ, Amaral L (1973). Identification and partial purification of "transcortin"-like protein within human lymphocytes. J Biol Chem.

[B117] Fernandez-Real JM, Pugeat M, Grasa M, Broch M, Vendrell J, Brun J, Ricart W (2002). Serum corticosteroid-binding globulin concentration and insulin resistance syndrome: a population study. J Clin Endocrinol Metab.

[B118] Ousova O, Guyonnet-Duperat V, Iannuccelli N, Bidanel JP, Milan D, Genet C, Llamas B, Yerle M, Gellin J, Chardon P, Emptoz-Bonneton A, Pugeat M, Mormede P, Moisan MP (2004). Corticosteroid binding globulin: a new target for cortisol-driven obesity. Mol Endocrinol.

[B119] Torpy DJ, Bachmann AW, Grice JE, Fitzgerald SP, Phillips PJ, Whitworth JA, Jackson RV (2001). Familial corticosteroid-binding globulin deficiency due to a novel null mutation: association with fatigue and relative hypotension. J Clin Endocrinol Metab.

[B120] Emptoz-Bonneton A, Cousin P, Seguchi K, Avvakumov GV, Bully C, Hammond GL, Pugeat M (2000). Novel human corticosteroid-binding globulin variant with low cortisol-binding affinity. J Clin Endocrinol Metab.

[B121] Joyner JM, Hutley LJ, Bachmann AW, Torpy DJ, Prins JB (2003). Greater replication and differentiation of preadipocytes in inherited corticosteroid-binding globulin deficiency. Am J Physiol Endocrinol Metab.

[B122] Grasa MM, Cabot C, Fernandez-Lopez JA, Remesar X, Alemany M (2001). Modulation of corticosterone availability to white adipose tissue of lean and obese Zucker rats by corticosteroid-binding globulin. Horm Metab Res.

[B123] Tannin GM, Agarwal AK, Monder C, New MI, White PC (1991). The human gene for 11β-hydroxysteroid dehydrogenase. Structure, tissue distribution, and chromosomal localization. J Biol Chem.

[B124] Ozols J (1995). Lumenal orientation and post-translational modifications of the liver microsomal 11β-hydroxysteroid dehydrogenase. J Biol Chem.

[B125] Odermatt A, Arnold P, Stauffer A, Frey BM, Frey FJ (1999). The N-terminal anchor sequences of 11β-hydroxysteroid dehydrogenases determine their orientation in the endoplasmic reticulum membrane. J Biol Chem.

[B126] Kotelevtsev Y, Brown RW, Fleming S, Kenyon C, Edwards CR, Seckl JR, Mullins JJ (1999). Hypertension in mice lacking 11β-hydroxysteroid dehydrogenase type 2. J Clin Invest.

[B127] Liu Y, Nakagawa Y, Wang Y, Li R, Li X, Ohzeki T, Friedman TC (2003). Leptin activation of corticosterone production in hepatocytes may contribute to the reversal of obesity and hyperglycemia in leptin-deficient *ob/ob *mice. Diabetes.

[B128] Aoki K, Homma M, Hirano T, Oka K, Satoh S, Mukasa K, Ito S, Sekihara H (2001). mRNA and enzyme activity of hepatic 11β-hydroxysteroid dehydrogenase type 1 are elevated in C57BL/KsJ-*db/db *mice. Life Sci.

[B129] Livingstone DE, Kenyon CJ, Walker BR (2000). Mechanisms of dysregulation of 11β-hydroxysteroid dehydrogenase type 1 in obese Zucker rats. J Endocrinol.

[B130] Livingstone DE, Jones GC, Smith K, Jamieson PM, Andrew R, Kenyon CJ, Walker BR (2000). Understanding the role of glucocorticoids in obesity: tissue-specific alterations of corticosterone metabolism in obese Zucker rats. Endocrinology.

[B131] Rask E, Olsson T, Soderberg S, Andrew R, Livingstone DE, Johnson O, Walker BR (2001). Tissue-specific dysregulation of cortisol metabolism in human obesity. J Clin Endocrinol Metab.

[B132] Stewart PM, Boulton A, Kumar S, Clark PM, Shackleton CH (1999). Cortisol metabolism in human obesity: impaired cortisone-->cortisol conversion in subjects with central adiposity. J Clin Endocrinol Metab.

[B133] Rask E, Walker BR, Soderberg S, Livingstone DE, Eliasson M, Johnson O, Andrew R, Olsson T (2002). Tissue-specific changes in peripheral cortisol metabolism in obese women: increased adipose 11β-hydroxysteroid dehydrogenase type 1 activity. J Clin Endocrinol Metab.

[B134] Wake DJ, Rask E, Livingstone DE, Soderberg S, Olsson T, Walker BR (2003). Local and systemic impact of transcriptional up-regulation of 11β-hydroxysteroid dehydrogenase type 1 in adipose tissue in human obesity. J Clin Endocrinol Metab.

[B135] Valsamakis G, Anwar A, Tomlinson JW, Shackleton CH, McTernan PG, Chetty R, Wood PJ, Banerjee AK, Holder G, Barnett AH, Stewart PM, Kumar S (2004). 11β-hydroxysteroid dehydrogenase type 1 activity in lean and obese males with type 2 diabetes mellitus. J Clin Endocrinol Metab.

[B136] Paulmyer-Lacroix O, Boullu S, Oliver C, Alessi MC, Grino M (2002). Expression of the mRNA coding for 11β-hydroxysteroid dehydrogenase type 1 in adipose tissue from obese patients: an in situ hybridization study. J Clin Endocrinol Metab.

[B137] Lindsay RS, Wake DJ, Nair S, Bunt J, Livingstone DE, Permana PA, Tataranni PA, Walker BR (2003). Subcutaneous adipose 11β-hydroxysteroid dehydrogenase type 1 activity and messenger ribonucleic acid levels are associated with adiposity and insulinemia in Pima Indians and Caucasians. J Clin Endocrinol Metab.

[B138] Kannisto K, Pietilainen KH, Ehrenborg E, Rissanen A, Kaprio J, Hamsten A, Yki-Jarvinen H (2004). Overexpression of 11β-hydroxysteroid dehydrogenase-1 in adipose tissue is associated with acquired obesity and features of insulin resistance: studies in young adult monozygotic twins. J Clin Endocrinol Metab.

[B139] Bujalska IJ, Kumar S, Stewart PM (1997). Does central obesity reflect "Cushing's disease of the omentum"?. Lancet.

[B140] Tomlinson JW, Sinha B, Bujalska I, Hewison M, Stewart PM (2002). Expression of 11β-hydroxysteroid dehydrogenase type 1 in adipose tissue is not increased in human obesity. J Clin Endocrinol Metab.

[B141] Livingstone DE, Walker BR (2003). Is 11β-hydroxysteroid dehydrogenase type 1 a therapeutic target? Effects of carbenoxolone in lean and obese Zucker rats. J Pharmacol Exp Ther.

[B142] Carr A, Cooper DA (2000). Adverse effects of antiretroviral therapy. Lancet.

[B143] Miller KK, Daly PA, Sentochnik D, Doweiko J, Samore M, Basgoz NO, Grinspoon SK (1998). Pseudo-Cushing's syndrome in human immunodeficiency virus-infected patients. Clin Infect Dis.

[B144] Sutinen J, Kannisto K, Korsheninnikova E, Nyman T, Ehrenborg E, Andrew R, Wake DJ, Hamsten A, Walker BR, Yki-Jarvinen H (2004). In the lipodystrophy associated with highly active antiretroviral therapy, pseudo-Cushing's syndrome is associated with increased regeneration of cortisol by 11beta-hydroxysteroid dehydrogenase type 1 in adipose tissue. Diabetologia.

[B145] Kotelevtsev Y, Holmes MC, Burchell A, Houston PM, Schmoll D, Jamieson P, Best R, Brown R, Edwards CR, Seckl JR, Mullins JJ (1997). 11β-hydroxysteroid dehydrogenase type 1 knockout mice show attenuated glucocorticoid-inducible responses and resist hyperglycemia on obesity or stress. Proc Natl Acad Sci USA.

[B146] Morton NM, Paterson JM, Masuzaki H, Holmes MC, Staels B, Fievet C, Walker BR, Flier JS, Mullins JJ, Seckl JR (2004). Novel adipose tissue-mediated resistance to diet-induced visceral obesity in 11β-hydroxysteroid dehydrogenase type 1-deficient mice. Diabetes.

[B147] Morton NM, Holmes MC, Fievet C, Staels B, Tailleux A, Mullins JJ, Seckl JR (2001). Improved lipid and lipoprotein profile, hepatic insulin sensitivity, and glucose tolerance in 11β-hydroxysteroid dehydrogenase type 1 null mice. J Biol Chem.

[B148] Justesen J, Mosekilde L, Holmes M, Stenderup K, Gasser J, Mullins JJ, Seckl JR, Kassem M (2004). Mice deficient in 11β-hydroxysteroid dehydrogenase type 1 lack bone marrow adipocytes, but maintain normal bone formation. Endocrinology.

[B149] Harris HJ, Kotelevtsev Y, Mullins JJ, Seckl JR, Holmes MC (2001). Intracellular regeneration of glucocorticoids by 11β-hydroxysteroid dehydrogenase (11β-HSD)-1 plays a key role in regulation of the hypothalamic-pituitary-adrenal axis: analysis of 11β-HSD-1-deficient mice. Endocrinology.

[B150] Yau JL, Noble J, Kenyon CJ, Hibberd C, Kotelevtsev Y, Mullins JJ, Seckl JR (2001). Lack of tissue glucocorticoid reactivation in 11β-hydroxysteroid dehydrogenase type 1 knockout mice ameliorates age-related learning impairments. Proc Natl Acad Sci USA.

[B151] Masuzaki H, Paterson J, Shinyama H, Morton NM, Mullins JJ, Seckl JR, Flier JS (2001). A transgenic model of visceral obesity and the metabolic syndrome. Science.

[B152] Masuzaki H, Yamamoto H, Kenyon CJ, Elmquist JK, Morton NM, Paterson JM, Shinyama H, Sharp MG, Fleming S, Mullins JJ, Seckl JR, Flier JS (2003). Transgenic amplification of glucocorticoid action in adipose tissue causes high blood pressure in mice. J Clin Invest.

[B153] Paterson JM, Morton NM, Fievet C, Kenyon CJ, Holmes MC, Staels B, Seckl JR, Mullins JJ (2004). Metabolic syndrome without obesity: Hepatic overexpression of 11β-hydroxysteroid dehydrogenase type 1 in transgenic mice. Proc Natl Acad Sci USA.

[B154] Phillipou G, Higgins BA (1985). A new defect in the peripheral conversion of cortisone to cortisol. J Steroid Biochem.

[B155] Jamieson A, Wallace AM, Andrew R, Nunez BS, Walker BR, Fraser R, White PC, Connell JM (1999). Apparent cortisone reductase deficiency: a functional defect in 11β-hydroxysteroid dehydrogenase type 1. J Clin Endocrinol Metab.

[B156] Biason-Lauber A, Suter SL, Shackleton CH, Zachmann M (2000). Apparent cortisone reductase deficiency: a rare cause of hyperandrogenemia and hypercortisolism. Horm Res.

[B157] Tomlinson JW, Draper N, Mackie J, Johnson AP, Holder G, Wood P, Stewart PM (2002). Absence of Cushingoid phenotype in a patient with Cushing's disease due to defective cortisone to cortisol conversion. J Clin Endocrinol Metab.

[B158] Malunowicz EM, Romer TE, Urban M, Bossowski A (2003). 11β-hydroxysteroid dehydrogenase type 1 deficiency ('apparent cortisone reductase deficiency') in a 6-year-old boy. Horm Res.

[B159] Draper N, Echwald SM, Lavery GG, Walker EA, Fraser R, Davies E, Sorensen TI, Astrup A, Adamski J, Hewison M, Connell JM, Pedersen O, Stewart PM (2002). Association studies between microsatellite markers within the gene encoding human 11β-hydroxysteroid dehydrogenase type 1 and body mass index, waist to hip ratio, and glucocorticoid metabolism. J Clin Endocrinol Metab.

[B160] Gelernter-Yaniv L, Feng N, Sebring NG, Hochberg Z, Yanovski JA (2003). Associations between a polymorphism in the 11β-hydroxysteroid dehydrogenase type I gene and body composition. Int J Obes Relat Metab Disord.

[B161] Davani B, Khan A, Hult M, Martensson E, Okret S, Efendic S, Jornvall H, Oppermann UC (2000). Type 1 11β-hydroxysteroid dehydrogenase mediates glucocorticoid activation and insulin release in pancreatic islets. J Biol Chem.

[B162] Duplomb L, Lee Y, Wang MY, Park BH, Takaishi K, Agarwal AK, Unger RH (2004). Increased expression and activity of 11β-HSD-1 in diabetic islets and prevention with troglitazone. Biochem Biophys Res Commun.

[B163] Sampath-Kumar R, Yu M, Khalil MW, Yang K (1997). Metyrapone is a competitive inhibitor of 11β-hydroxysteroid dehydrogenase type 1 reductase. J Steroid Biochem Mol Biol.

[B164] Hult M, Jornvall H, Oppermann UC (1998). Selective inhibition of human type 1 11β-hydroxysteroid dehydrogenase by synthetic steroids and xenobiotics. FEBS Lett.

[B165] Diederich S, Grossmann C, Hanke B, Quinkler M, Herrmann M, Bahr V, Oelkers W (2000). In the search for specific inhibitors of human 11β-hydroxysteroid-dehydrogenases (11β-HSDs): chenodeoxycholic acid selectively inhibits 11β-HSD-I. Eur J Endocrinol.

[B166] Stauffer AT, Rochat MK, Dick B, Frey FJ, Odermatt A (2002). Chenodeoxycholic acid and deoxycholic acid inhibit 11β-hydroxysteroid dehydrogenase type 2 and cause cortisol-induced transcriptional activation of the mineralocorticoid receptor. J Biol Chem.

[B167] Morris DJ, Souness GW, Latif SA, Hardy MP, Brem AS (2004). Effect of chenodeoxycholic acid on 11β-hydroxysteroid dehydrogenase in various target tissues. Metabolism.

[B168] Andrews RC, Rooyackers O, Walker BR (2003). Effects of the 11β-hydroxysteroid dehydrogenase inhibitor carbenoxolone on insulin sensitivity in men with type 2 diabetes. J Clin Endocrinol Metab.

[B169] Sandeep TC, Yau JL, MacLullich AM, Noble J, Deary IJ, Walker BR, Seckl JR (2004). 11β-hydroxysteroid dehydrogenase inhibition improves cognitive function in healthy elderly men and type 2 diabetics. Proc Natl Acad Sci USA.

[B170] Barf T, Vallgarda J, Emond R, Haggstrom C, Kurz G, Nygren A, Larwood V, Mosialou E, Axelsson K, Olsson R, Engblom L, Edling N, Ronquist-Nii Y, Ohman B, Alberts P, Abrahmsen L (2002). Arylsulfonamidothiazoles as a new class of potential antidiabetic drugs: Discovery of potent and selective inhibitors of the 11β-hydroxysteroid dehydrogenase type 1. J Med Chem.

[B171] Alberts P, Engblom L, Edling N, Forsgren M, Klingstrom G, Larsson C, Ronquist-Nii Y, Ohman B, Abrahmsen L (2002). Selective inhibition of 11β-hydroxysteroid dehydrogenase type 1 decreases blood glucose concentrations in hyperglycaemic mice. Diabetologia.

[B172] Alberts P, Nilsson C, Selen G, Engblom LO, Edling NH, Norling S, Klingstrom G, Larsson C, Forsgren M, Ashkzari M, Nilsson CE, Fiedler M, Bergqvist E, Ohman B, Bjorkstrand E, Abrahmsen LB (2003). Selective inhibition of 11β-hydroxysteroid dehydrogenase type 1 improves hepatic insulin sensitivity in hyperglycemic mice strains. Endocrinology.

[B173] Hermanowski-Vosatka A, Mundt S, Nunes C, Strowski M, Li Z, Frazier E, Le Grand C, Chen H, Robertson N, Metzger J, Strack A, Olson S, Schaeffer J, Wright SD, Balkovec J, Thieringer R (2004). 11β-HSD1 inhibition ameliorates metabolic syndrome in mice. The Keystone Symposia for Adipogenesis and Obesity (X2) 2004 Abstract Book: 4–10 March 2004; Banff, Alberta, Canada.

[B174] Friedman JE, Sun Y, Ishizuka T, Farrell CJ, McCormack SE, Herron LM, Hakimi P, Lechner P, Yun JS (1997). Phosphoenolpyruvate carboxykinase (GTP) gene transcription and hyperglycemia are regulated by glucocorticoids in genetically obese *db/db *transgenic mice. J Biol Chem.

[B175] Hundal RS, Krssak M, Dufour S, Laurent D, Lebon V, Chandramouli V, Inzucchi SE, Schumann WC, Petersen KF, Landau BR, Shulman GI (2000). Mechanism by which metformin reduces glucose production in type 2 diabetes. Diabetes.

[B176] Gruol DJ, Altschmied J (1993). Synergistic induction of apoptosis with glucocorticoids and 3',5'-cyclic adenosine monophosphate reveals agonist activity by RU 486. Mol Endocrinol.

[B177] Garrel DR, Moussali R, De Oliveira A, Lesiege D, Lariviere F (1995). RU 486 prevents the acute effects of cortisol on glucose and leucine metabolism. J Clin Endocrinol Metab.

[B178] Lamberts SW, Koper JW, de Jong FH (1991). The endocrine effects of long-term treatment with mifepristone (RU 486). J Clin Endocrinol Metab.

[B179] Kim PJ, Cole MA, Kalman BA, Spencer RL (1998). Evaluation of RU28318 and RU40555 as selective mineralocorticoid receptor and glucocorticoid receptor antagonists, respectively: receptor measures and functional studies. J Steroid Biochem Mol Biol.

[B180] von Geldern TW, Tu N, Kym PR, Link JT, Jae HS, Lai C, Apelqvist T, Rhonnstad P, Hagberg L, Koehler K, Grynfarb M, Goos-Nilsson A, Sandberg J, Osterlund M, Barkhem T, Hoglund M, Wang J, Fung S, Wilcox D, Nguyen P, Jakob C, Hutchins C, Farnegardh M, Kauppi B, Ohman L, Jacobson PB (2004). Liver-selective glucocorticoid antagonists: a novel treatment for type 2 diabetes. J Med Chem.

